# Screening Potential Diagnostic Biomarkers of Lung Adenocarcinoma Related to Immune Infiltration Based on Single‐Cell Proteomic Integration Analysis

**DOI:** 10.1155/carj/7004493

**Published:** 2026-08-17

**Authors:** Ningxin Xu, Yuwei Zhang, Qiongke Lian, Yanan Cai, Mingli Ni

**Affiliations:** ^1^ Department of Oncology, Luoyang Central Hospital Affiliated to Zhengzhou University, Luoyang 471000, Henan, China, zzu.edu.cn

**Keywords:** lung adenocarcinoma, macrophages, proteomics, single-cell sequencing

## Abstract

**Background:**

Macrophages play a crucial role in mediating immune infiltration in lung adenocarcinoma (LUAD). However, the potential of macrophage‐related targets in the diagnosis and treatment of LUAD remains largely unexplored.

**Methods:**

The proteome data of LUAD were obtained from the CPTAC database, and the single‐cell sequencing (scRNA‐seq) data of non–small‐cell lung cancer (NSCLC) were collected from the GEO database. The differentially expressed proteins (DEPs) of LUAD and module proteins closely associated with clinical features were analyzed by the limma tool and WGCNA. The 61 intersection targets of DEPs and WGCNA were obtained for GO and KEGG pathway enrichment analysis. Machine learning (random forest algorithm, LASSO regression analysis, and support vector machine recursive feature elimination algorithm) approaches screened the hub targets related to macrophages in LUAD. The CIBERSORT method and GSEA were used to explore the connection between key targets and immune cells. ROC curve was used to construct genetic diagnosis model, and the potential targeted drugs of four hub genes were screened by DSigDB database.

**Results:**

A total of 1149 DEPs in LUAD and 65 proteins associated with clinical features were screened. After the intersection, 61 targets were obtained for GO and KEGG analysis. Macrophages were the key cell type in LUAD, which had 5438 differentially expressed genes in LUAD. Moreover, 13 intersection targets were obtained by taking the intersection of proteomes (61 targets) and macrophage‐related genes (5438 genes). And seven targets (CRYAB, EHD2, FHL1, TNS1, TNXB, SORBS2, and LTBP4) were obtained through machine learning. Based on CIBERSORT method and GSEA enrichment results, four genes (TNS1, EHD2, CRYAB, and TNXB) related to immune infiltration were screened. ROC analysis combined with the nomogram‐related model showed that only four hub genes (EHD2, FHL1, TNS1, and SORBS2) could be used as diagnostic biomarkers, and their potential therapeutic drugs were revealed.

**Conclusion:**

This study identified four hub targets, which have certain value in the diagnosis and the development of targeted drugs for LUAD.

## 1. Introduction

Non–small‐cell lung cancer (NSCLC) accounts for more than 85% of cases of lung cancer, of which the main subtypes are lung adenocarcinoma (LUAD) and lung squamous cell carcinoma (LUSC) [1–3]. Despite the progress of targeted therapy and immunotherapy in recent years, the difficulty of early diagnosis is still a key factor leading to poor prognosis in LUAD patients [4]. Traditional diagnostic methods such as imaging examination and tissue biopsy have limitations such as low sensitivity and strong invasiveness. Therefore, there is an urgent need to develop highly specific molecular markers to achieve early screening and accurate diagnosis for LUAD.

Immune infiltration in the tumor microenvironment (TME) is closely related to cancer development, and its molecular mechanism involves a complex protein regulatory network [5, 6]. Macrophages, as the core component of TME, affect the development and metastasis of tumors through many mechanisms [7]. Studies have shown that macrophages can secrete immunosuppressive factors to inhibit the activity of T cells and NK cells and express immune checkpoint molecules to help tumors evade immune surveillance [8–10]. Also, macrophages can also secrete growth factors, induce angiogenesis, stimulate tumor cell growth, and inhibit apoptosis [11, 12]. In addition, macrophages compete with tumor cells for resources through aerobic glycolysis or oxidative metabolism, which alters TME and then affects the metabolism of tumor cells [13, 14]. Therefore, analysis of the key genes that affect the function of macrophages may provide new ideas for overcoming the immune infiltration of LUAD cells and developing potential biomarkers.

Proteomics is a large‐scale study of all proteins expressed in an organism, tissue, or cell at a specific time and under a specific condition, which can identify cancer‐related proteins by comparing the proteome of tumor and normal tissues [15–19]. Single‐cell sequencing (scRNA‐seq) is to sequence the genome, transcriptome, and epigenome at the single‐cell level to analyze the heterogeneity between cells [20]. The combination of proteomics technology and scRNA‐seq provides a new perspective for analyzing tumor heterogeneity and screening key markers [21, 22]. Based on the proteome data of LUAD from CPTAC database and scRNA‐seq data of NSCLC from GEO database, the aim of this study is to screen key targets related to immune infiltration and construct a diagnostic model, thus providing potential biomarkers for LUAD.

## 2. Materials and Methods

### 2.1. Identification of Differentially Expressed Proteins (DEPs) in LUAD Tissues

Proteome analysis data of LUAD (PDC Study ID: PDC000153, including 110 LUAD tumor tissues and 101 normal tissues) were obtained from CPTAC database (https://pdc.cancer.gov/pdc/browse). PDC000153 dataset was used for differential protein screening, WGCNA analysis, machine learning modeling, the first version of ROC construction and internal evaluation, which served as the modeling training data. After removing the proteins containing missing values, a limma rapid differential analysis tool (version 3.40.6) of sangerbox was used to analyze the DEPs. Specifically, the acquired expression profiling data sets were subjected to multiple linear regression using the lmFit function, and eBays function was further used to compute moderated *t*‐statistics, moderated *F*‐statistic, and log‐odds of differential expression. The difference significance of each protein was obtained (threshold screening: *p* < 0.05 and |log_2_FoldChange| > 0.585).

### 2.2. Identification of Proteins That Highly Associated With Clinical Features

The median absolute deviation (MAD) of each protein was calculated using LUAD expression profiles (PDC Study ID: PDC000153, 106 samples with survival time were selected), and the top 50% of genes with the smallest MAD were removed. Outlier genes and samples were removed by goodSamplesGenes method of R package WGCNA, and WGCNA was further used to construct scale‐free co‐expression network. To further analyze the module, the dissimilarity of module eight genes was calculated, and a cut line for the module dendrogram was chosen and some modules were merged. In addition, modules with a distance less than 0.25 were merged, and 14 co‐expression modules were finally obtained.

### 2.3. GO and KEGG Analysis

The Venn diagram was used to analyze the 61 key targets by taking the intersection of DEPs and WGCNA. The protein–protein interaction (PPI) network of the 61 intersected targets was displayed by the STRING website (https://cn.string-db.org/) and optimized using Cytoscape_v3.9.1. The obtained 61 intersection targets were input into the DAVID database (https://david.ncifcrf.gov/) for GO and KEGG pathway analyses. The TOP10 *p* values were selected to draw the corresponding enriched Sankey‐bubble chart through the sangerbox platform (threshold screening: *p* < 0.05).

### 2.4. Immune Cell Subsets Analysis

The sangerbox analysis with the CIBERSORT method was used to calculate the immune microenvironment score in LUAD expression data. The relative proportions of 22 immune cell subsets in 211 tissue samples are shown as multiple‐group stacked bar graphs. Spearman correlation analysis was used to evaluate the correlations between 22 immune cell subsets.

### 2.5. scRNA‐seq Analyzed Macrophage‐Related Genes

The scRNA‐seq data of NSCLC (accession: GSE117570, including three LUAD samples and one LUSC sample) was downloaded from the GEO database (https://www.ncbi.nlm.nih.gov/geo/). Quality control standard: abnormal cells with a single‐cell gene count of less than 200 or more than 5000 were eliminated, and ultimately 4533 qualified cells were retained for subsequent analysis. The clustering parameters are listed as follows: the Seurat standard process is adopted; PCA is used for dimensionality reduction, and the Harmony package is used to remove the batch effect. Based on the first 20 principal components, UMAP dimension reduction is performed, and the clustering resolution of FindClusters is set to 0.1. Cell type annotation criteria: cell types were defined strictly according to classic marker genes in CellMarker database: Macrophages (MSR1/CD68/MARCO), T cells (CD7/BATF), B cells (IGHA1/IGHG1/IGHM), epithelial cells (EPCAM/KRT18/19), endothelial cells (IGFBP7/TCF4/KLF9). Differential genes associated with macrophages were screened by differential analysis between cell types, and presented as volcano plots (1291 upregulated and 4147 downregulated).

### 2.6. Hub Genes Related to Macrophages in LUAD Patients

The Venn diagram analyzed the intersection of genes of macrophage‐associated genes (5438) and proteome‐differential proteins (61). The LASSO algorithm is suitable for high‐dimensional data, which can compress variables and avoid multicollinearity; support vector machine recursive feature elimination (SVM‐RFE) algorithm is good at screening the strong discriminative features of the binary diagnostic model. Random forest (RF) algorithm ensemble learning assesses gene importance and reduces single‐algorithm error. The combination of the three ensures that the core target screening is stable and reproducible. Cross‐validation: 5‐fold cross‐validation was used: SVM‐RFE: the accuracy of cross‐validation was the highest (0.995) when 10 features were selected; LASSO: 9 candidate genes were selected under the optimal *λ* value; RF: outputting the most important TOP10 genes. Finally, the intersection of the three factors was used to determine the hub genes to ensure the robustness of the screening results.

### 2.7. Immune Infiltration and GSEA Analysis

The sangerbox analysis with the CIBERSORT method was used to explore the connection between key targets and immune cells in LUAD expression data. The GSEA website (https://software.broadinstitute.org/gsea/index.jsp) was used to download the GSEA software (Version 3.0). Samples were divided into a high‐expression group (≥ 50%) and low‐expression group (< 50%) according to the expression levels of the seven genes. The c2.cp.kegg.v 7.4.symbols.gmt was obtained from the Molecular Signatures Database (https://www.gsea-msigdb.org/gsea/downloads.jsp) to assess the pathways and molecular mechanisms involved. Based on the gene expression profile and phenotype grouping, the minimum gene set is 5, the maximum gene set is 5000, with 1000 resampling. *p* < 0.05, |Normalized Enrichment Score (NES)| > 1, and False Discovery Rate (FDR) < 0.3 were considered statistically significant. A larger value of |NES| leads to a smaller FDR value, indicating that the results of the analysis are more credible. NES plus and minus TOP5 pathways were selected for display.

### 2.8. Genetic Diagnosis Model

ROC analysis of the data was performed using the pROC package, and the results were visualized with ggplot2. The ROC curve was used to reflect the relationship between sensitivity and specificity. After data cleaning, a binary logistic model was constructed using the glm function, and a nomogram correlation model was constructed and visualized using the rms package. PDC000234 (LUSC proteome cohort, including 110 tumor tissues and 102 normal tissues) was an independent external validation dataset. This dataset was not involved in any model training, feature screening, and parameter debugging at all. It was only used as a completely independent blind external test set to verify the generalization ability of the four‐gene diagnostic model and strictly avoid the bias caused by data leakage. When constructing the multivariate logistic diagnostic model, genes with extreme deviation and multicollinearity (CRYAB, TNXB, and LTBP4) were excluded in advance to avoid redundant variables that would increase the fitting accuracy and cause high apparent AUC but poor actual generalization. Finally, the test dataset (PDC Study ID: PDC000153) and validation dataset (PDC Study ID: PDC000234) were used to analyze the diagnostic ROC curves of the logistic regression model for 4 hub genes (EHD2, FHL1, TNS1 and SORBS2).

### 2.9. Candidate Drugs of Key Target

According to the *p* value < 0.05 set by DSigDB database, the potential targeted drugs of four targets (EHD2, FHL1, TNS1, and SORBS2) were screened, and the TOP10 candidate drugs are shown in Table 1.

**Table 1:** Candidate drugs predicted using DSigDB.

Term	*p* value	Combined score	Genes
estradiol CTD 00005920	0.002206777	383218.0053	EHD2; FHL1; SORBS2; TNS1
etoposide CTD 00005948	0.003084494	246.0796934	FHL1; SORBS2
diphenylpyraline PC3 DOWN	0.004990939	1470.197502	FHL1
cicloheximide HL60 DOWN	0.006693256	141.7867023	FHL1; TNS1
ketoconazole HL60 DOWN	0.007578847	877.9072583	TNS1
Lidocaine hydrochloride BOSS	0.007578847	877.9072583	TNS1
lidocaine PC3 DOWN	0.007777708	850.2271896	FHL1
sulfaguanidine PC3 UP	0.009285848	111.2644256	EHD2; TNS1
betulinic acid HL60 DOWN	0.009367518	675.1928734	TNS1
progesterone HL60 DOWN	0.009764672	641.2429069	TNS1

## 3. Results

### 3.1. Screening of DEPs in LUAD Tumor Tissues and Normal Tissues

**FIGURE 1:**
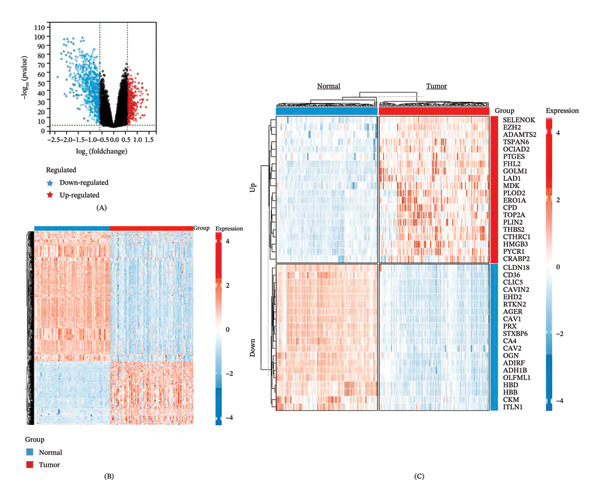
DEPs were screened in LUAD tumor tissues and normal tissues. (A) Volcano plot showed the upregulated and downregulated proteins in LUAD tumor tissues. (B) Heat map exhibited the DEPs in LUAD tumor tissues and normal tissues. (C) Heat map showed the TOP20 upregulated and downregulated proteins in LUAD tumor tissues and normal tissues.

The limma analysis tool of sangerbox was used to analyze the DEPs in LUAD tumor tissues and normal tissues. The volcano plot showed a total of 376 upregulated and 773 downregulated proteins in LUAD tumor tissues (Figure 1A). The heat map of all identified DEPs is shown in (Figure 1B), and the heat map of TOP20 upregulated and downregulated proteins is presented in (Figure 1C).

### 3.2. WGCNA Identified Proteins That Highly Correlated With Clinical Features in LUAD

**FIGURE 2:**
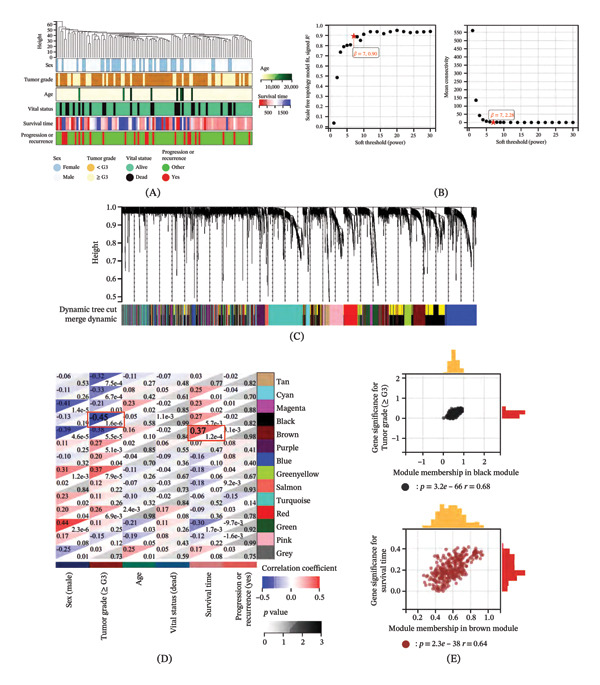
Identification of proteins that highly correlated with clinical features in LUAD through WGCNA. (A) Preliminary gene clustering in WGCNA. (B) The optimal soft‐thresholding power was determined to be 7. (C) A dynamic tree cut was visualized. (D) Heat map showed the correlation analysis between modules and features. (E) The module membership‐gene significance scatter plots.

Furthermore, this study hopes to obtain proteins that are highly correlated with clinical features in LUAD using WGCNA. First, a cluster dendrogram based on gene correlation was constructed (Figure 2A). When the soft threshold *β* = 7, the scale‐free topology model fit signed *R* ^2^ was greater than 0.85, and the mean connectivity was less than 200 (Figure 2B). After merging the original module at a dynamic cutting height of 0.25 with several minor modules, a total of 14 co‐expressed modules were obtained (Figure 2C). The Module‐Feature Correlation heat map showed that the 39 genes in the black module associated with tumor grade and 26 genes in the brown module associated with survival time were the modules with the largest absolute value of correlation coefficients (Figure 2D). The module membership‐gene significance scatter plots of black module of tumor grade and brown module of survival time are shown in (Figure 2E).

### 3.3. PPI Network and KEGG/GO Analysis of Module‐Related DEPs in LUAD

**FIGURE 3:**
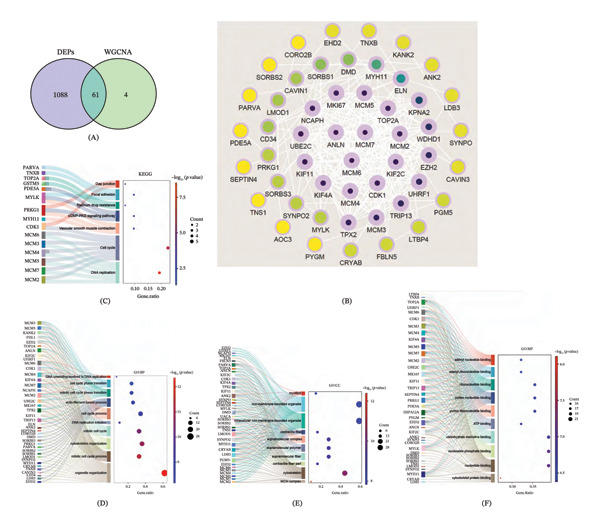
PPI network analysis and KEGG/GO analysis. (A) Venn diagram was used to screen the 61 intersection genes from DEPs and WGCNA. (B) The PPI network of 61 intersection proteins were visualized using the Cytoscape software. The functions of intersection proteins were analyzed by KEGG analysis (C) and GO analysis (D–F).

A total of 61 key proteins were obtained by taking the intersection of DEPs and the proteins screened by WGCNA using the Venn diagram (Figure 3A). The PPI network of 61 proteins analyzed by the STRING website and shown in (Figure 3B) after optimization using Cytoscape_v3.9.1. KEGG analysis revealed that these intersection proteins were involved in multiple pathway regulation, such as cell cycle, DNA replication, and vascular smooth muscle contraction (Figure 3C). GO analysis exhibited that the mainly biological process was organelle organization and mitotic cell cycle process (Figure 3D); the main cellular component was MCM complex and cytoskeleton (Figure 3E); the main molecular function was cytoskeletal protein binding and nucleotide binding (Figure 3F).

### 3.4. Analyzing the Immune Cell Subsets

Following, this study analyzed the immune cell subsets in LUAD. Multiple stacked bar charts showed the relative proportions of 22 immune cell subsets in all 211 tissues samples (Figure 4A). The heat map exhibited the correlation between the 22 immune cell subsets (Figure 4B). The differences in 22 immune cell subsets between normal tissues and LUAD tissues are shown in (Figure 4C).

FIGURE 4Immune cell subsets were analyzed. (A) The relative proportions of 22 immune cell subsets in all 211 tissues sample were analyzed by CIBERSORT. (B) Heat map was used to analyze the correlation between the 22 immune cell subsets. (C) The differences in 22 immune cell subsets between normal tissues and LUAD tissues were analyzed.

**Figure:**
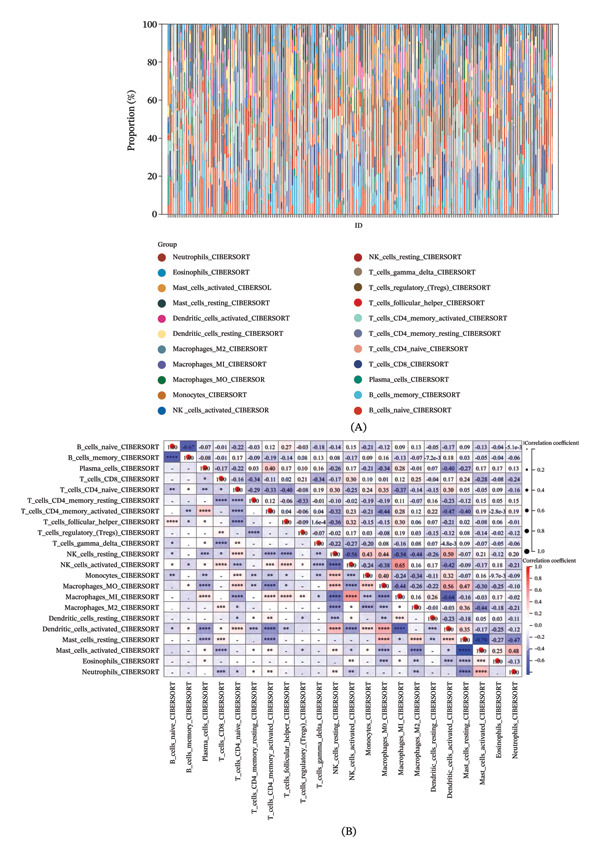

**Figure:**
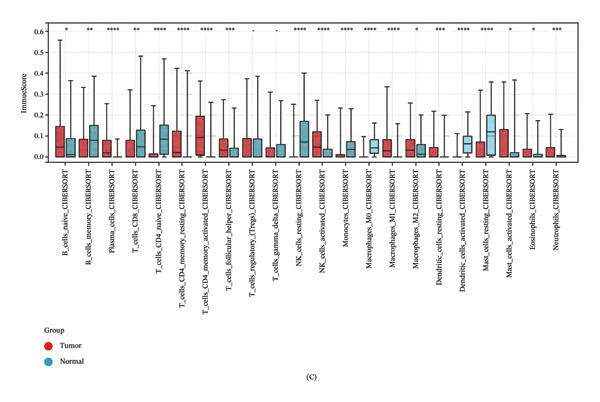

### 3.5. Analysis of Macrophage‐Related Genes Using scRNA‐Seq

scRNA‐seq data were used to analyze macrophage‐related genes. According to gene feature number, cell number count, and mitochondrial sequencing percentage, the high‐quality cells and genes from the scRNA‐seq of the GEO database (GSE117570) were screened (Figure 5A). Cluster analysis by UMAP algorithm identified 17 immune cell clusters in the GSE117570 dataset (Figure 5B), and the five identified cell types were macrophages, epithelial cells, T‐cells, B‐cells and endothelial cells (Figure 5C). Cell clusters were then annotated based on identified marker genes. The expression of typical marker genes of macrophages (MSR1, CD68, and MARCO), epithelial cells (EPCAM and KRT18/19), T‐cells (CD7 and BATF), B‐cells (IGHA1, IGHG1, and IGHM) and endothelial cells (IGFBP7, TCF4, and KLF9) was shown in (Figure 5D). The volcano plot identified 5438 differentially expressed genes (1291 upregulated and 4147 downregulated) associated with macrophage cells by differential analysis between cell types (Figure 5E), which were subjected to subsequent screening.

FIGURE 5scRNA‐seq screened the differential cells and markers in LUAD. (A) The screening of high‐quality cells and genes from scRNA‐seq. (B) UMAP algorithm was used to cluster analysis to identify 17 immune cell clusters. (C) UMAP algorithm analyzed the five identified cell types. (D) The expression of typical marker genes in five identified cell types is shown. (E) Volcano plot showed the differential expression of genes associated with macrophage cells.

**Figure:**
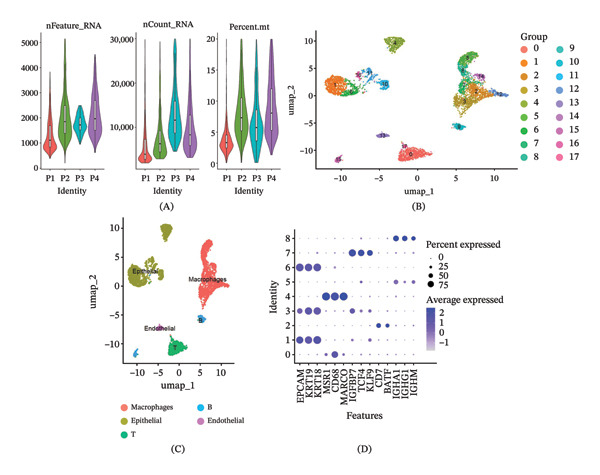

**Figure:**
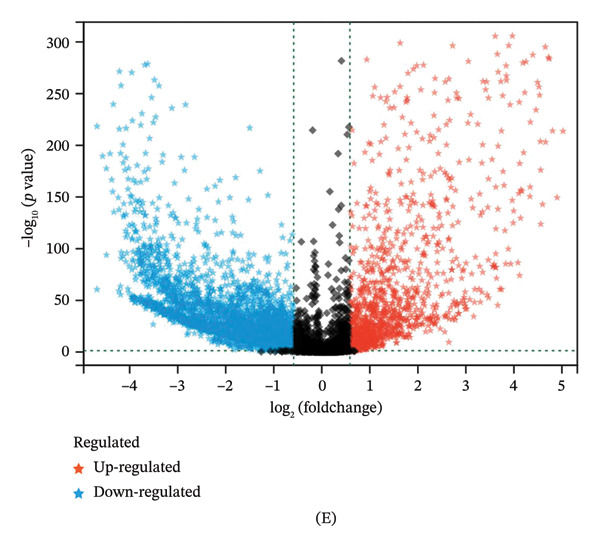

### 3.6. Machine Learning Approach Screened Hub Targets Related to Macrophages in LUAD

**FIGURE 6:**
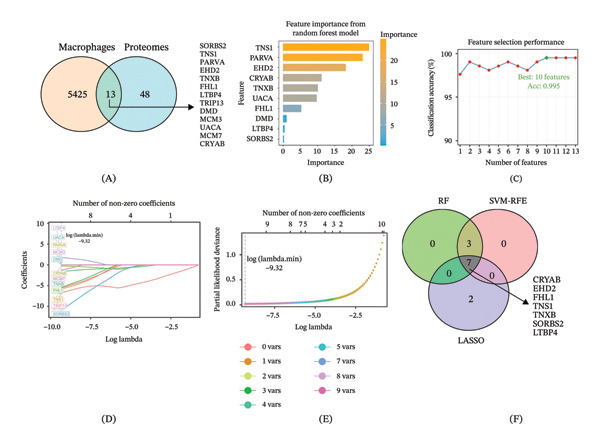
Hub targets related to macrophages in LUAD were screened. (A) Venn diagram showed the intersection targets of proteomes (61) and macrophage‐related genes (5438). RF algorithm (B), SVM‐RFE algorithm (C) and LASSO algorithm (D‐E) analyzed the candidate core targets. (F) Venn diagram analyzed the intersection targets according RF, SVM‐RFE and LASSO.

By taking the intersection of proteomes (61) and macrophage‐related genes (5438), a total of 13 intersection genes were obtained (Figure 6A). The variable importance graph shows the TOP10 genes that contribute the most to the model as determined by the RF algorithm (Figure 6B). The SVM‐RFE indicated that when the number of features was 10, the cross‐validation accuracy reached the highest value of 0.995, indicating that 10 features might be the best choice for the model (Figure 6C). The LASSO model showed that the number of genes identified that best fit the requirements for candidate core targets was 9 at the lowest point of the curve (Figure 6D‐E). Finally, seven intersection targets (CRYAB, EHD2, FHL1, TNS1, TNXB, SORBS2, and LTBP4) were obtained by taking the above three algorithms (Figure 6F).

### 3.7. Immune Infiltration and GSEA Analysis of 7 Genes

Following, this study analyzed the LUAD data to explore the association between key genes and immune cells using the CIBERSORT method. The correlations between the seven genes and the 22 immune cell subsets are shown in Figure 7A. In addition, the expression of 7 genes in the LUAD proteomes is shown in Figure 7B. After GSEA analysis of the 7 core genes, TNS1, EHD2, CRYAB, and TNXB were significantly enriched in the high/low expression group (FDR < 0.3) (Figure 7C). However, FHL1, SORBS2, and LTBP4 did not detect enriched pathways that met the above significance criteria and were therefore not included in the subsequent presentation.

FIGURE 7Immune infiltration and GSEA analysis. (A) Heat map was used to analyze the correlation between seven genes and 22 immune cell subsets. (B) The expression of seven genes in the proteome of LUAD tissues and normal tissues was shown. (C) GSEA was used to analyze the significantly enriched pathways of TNS1, EHD2, CRYAB, and TNXB (NES plus and minus TOP5 pathways were shown; H: negatively correlated; L: positively correlated).

**Figure:**
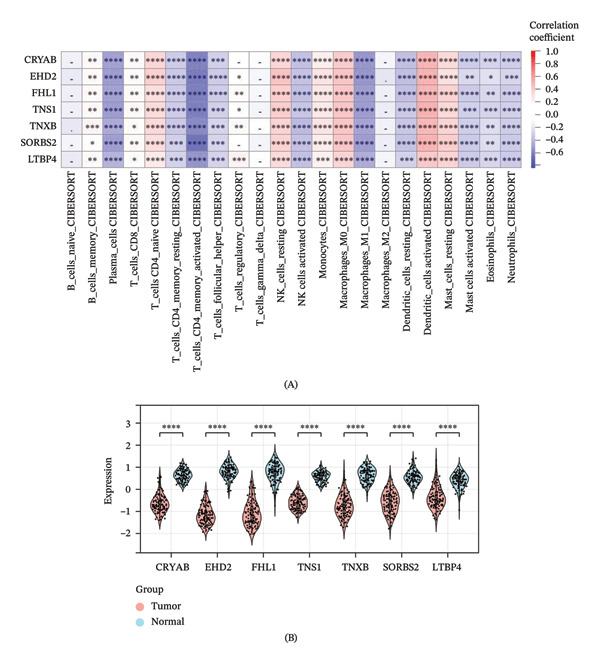

**Figure:**
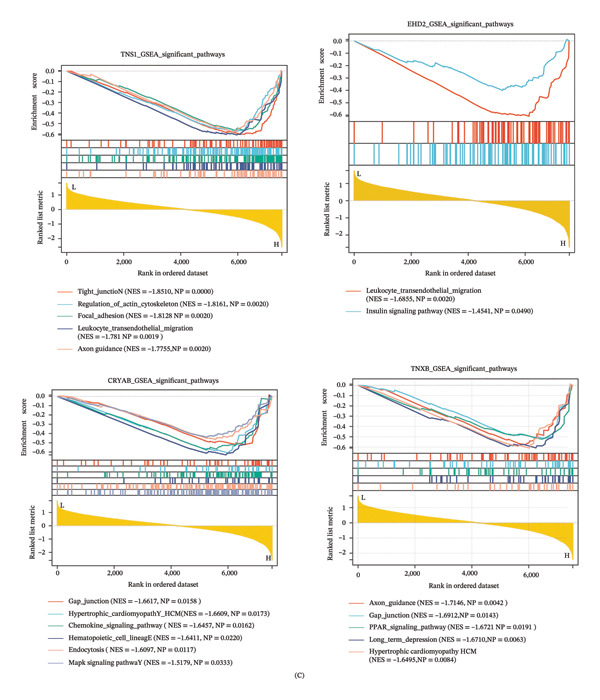

### 3.8. Construction of Genetic Diagnosis Models

**FIGURE 8:**
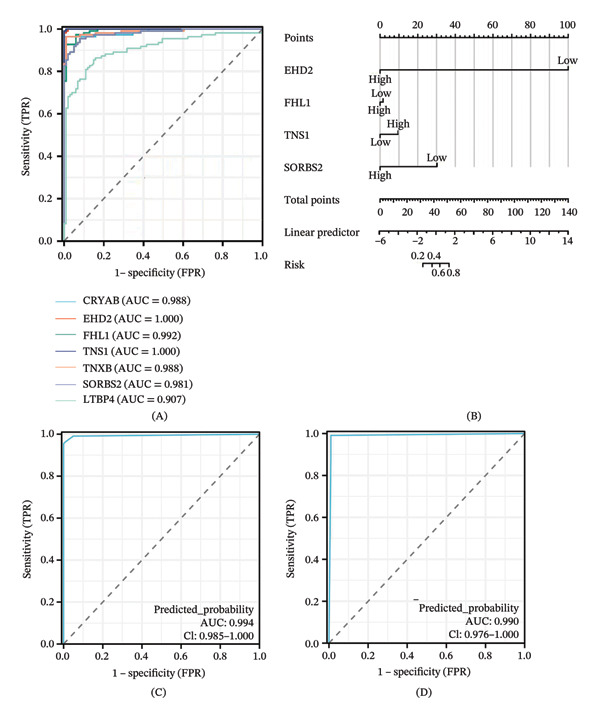
Genetic diagnosis models were constructed. (A) ROC curve assessed the diagnostic value of seven genes. (B) Nomogram‐related model analyzed four hub genes. ROC curves were used to analyze the diagnostic value for the four hub genes in test set (C) and validation set (D).

The pROC package was used for ROC analysis of the data, and the results showed that the seven genes had high accuracy, which could be used for the construction of diagnostic models (Figure 8A). Nomogram related model revealed that only 4 hub genes (EHD2, FHL1, TNS1, and SORBS2) were retained, while the remaining thrî genes (CRYAB, TNXB, and LTBP4) were eliminated due to predictive value or collinearity with other variables (Figure 8B). In the test set, the diagnostic ROC curves of the logistic regression model for the 4 hub genes are shown in Figure 8C. In the validation set (Proteomic data of LUSC, PDC Study ID: PDC000234), the diagnostic ROC curves of logistic regression models for the 4 hub genes are presented in Figure 8D. In addition, the expression of 4 hub genes in the LUAD proteomes is shown in Supporting Figure 1A, and the Nomogram of 4 hub genes in LUAD is exhibited in Supporting Figure. 1B.

### 3.9. Screening of Candidate Drugs for Key Genes

**FIGURE 9:**
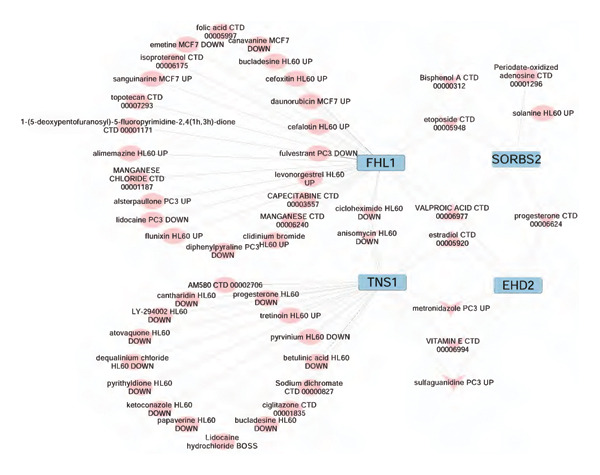
Candidate drugs for key genes were predicted. The DSigDB database predicted the potential targeted drugs for 4 hub genes.

According to the *p* < 0.05 set by the DSigDB database, the potential targeted drugs for 4 hub genes (EHD2, FHL1, TNS1, and SORBS2) were screened, as shown in Figure 9. And the TOP10 candidate drugs for EHD2, FHL1, TNS1, and SORBS2 are presented in Table 1.

## 4. Discussion

LUAD is a type of cancer that seriously threatens the lives of patients and brings great challenges to global medical care [23]. Therefore, the search for effective molecular targets is essential for early diagnosis and effective treatment strategies of LUAD [24, 25]. The relationship between immune cells in TME and cancer diagnosis, treatment, and prognosis has been extensively validated [26]. Thus, the development of genetic diagnostic models based on immune‐related molecules is a promising research direction for LUAD.

Proteomics is a scientific field that studies the expression, structure, function, and interaction of all proteins in living organisms [27]. In cancer research, proteomics provides an important tool for the mechanism exploration, early diagnosis, therapeutic target discovery, and personalized treatment of cancer by systematically analyzing the changes of tumor‐related proteins [28, 29]. Through proteomic analysis, Song and Yang found that the levels of 10 proteins were associated with the risk of breast cancer, among which 6 proteins had been identified as the potential therapeutic targets [30]. Wang et al. identified 5 proteins associated with lung cancer risk by proteomic analysis and experimentally confirmed the potential therapeutic relevance of ICAM5 and SFTPB [31]. In this study, by analyzing the LUAD proteomic data, a total of 1149 DEPs were screened in LUAD tissues and normal tissues. Further WGCNA identified a total of 65 proteins highly associated with clinical features (39 proteins with tumor grade and 26 proteins with survival time). By analyzing the 61 proteins after intersection, this study confirmed that vascular smooth muscle contraction, DNA replication, and cell cycle are the main pathways in which these intersection proteins are involved. Also, 61 intersection proteins are mainly enriched in the biological process such as organelle organization and mitotic cell cycle process; the cellular component including MCM complex and cytoskeleton; the main molecular function including cytoskeletal protein binding and nucleotide binding. The above information indicates that the screened 61 intersection proteins can regulate LUAD progression through multiple pathways and functions.

Immune cells play a dual role in cancer progression, of which macrophages, especially tumor‐associated macrophages, are key regulators [32]. Macrophages can be polarized into M1 or M2 phenotypes based on microenvironmental signals, and their plasticity and versatility make them important targets for cancer therapy [33]. Our results revealed a total of 22 immune cell subsets in LUAD tissues. Further scRNA‐seq data analysis showed that macrophages, B‐cells, epithelial cells, T‐cells, and endothelial cells were 5 cell types that had been identified in LUAD, among which macrophages had a total of 5438 differentially expressed genes. Through the intersection of 61 proteomic analysis targets and 5438 macrophage‐related genes, our study screened 13 candidate genes related to immune infiltration. Seven genes (CRYAB, EHD2, FHL1, TNS1, TNXB, SORBS2, and LTBP4) were selected as core targets by LASSO regression analysis, SVM‐RFE, and the RF regression machine learning algorithm. After the CIBERSORT method and GSEA, four genes (TNS1, EHD2, CRYAB, and TNXB) were identified to be strongly associated with immune infiltration. Further ROC analysis showed that 4 genes (EHD2, TNS1, FHL1, and SORBS2) showed excellent diagnostic efficacy. In LUAD, the AUC of the combined model was 0.994 in the test cohort and 0.990 in the validation cohort, suggesting that they had broad diagnostic value for LUAD. In addition, TOP10 targeted drugs for four hub genes were screened by DSigDB database, including estradiol CTD 00005920, etoposide CTD 00005948, diphenylpyraline PC3 DOWN, cicloheximide HL60 DOWN, ketoconazole HL60 DOWN, Lidocaine hydrochloride BOSS, lidocaine PC3 DOWN, sulfaguanidine PC3 UP, betulinic acid HL60 DOWN, and progesterone HL60 DOWN, which provides directions for subsequent mechanism research and drug development in LUAD.

Our study has several limitations. First, all data were obtained from public databases (CPTAC and GEO) without independent external validation. Although an internal cohort was used for model validation, the lack of a prospective multicenter cohort limits the generalizability of our findings. Second, our study is the preliminary target mining, and key genes (EHD2, TNS1, FHL1, and SORBS2) were identified based on computational analysis but were not experimentally validated at the protein or functional level. Third, immune infiltration analysis relies on the CIBERSORT algorithm for inference rather than direct experimental measurements, and the functional role of macrophage‐related genes in the tumor immune microenvironment needs to be further elucidated by in vitro or in vivo studies. Therefore, in the future, it is necessary to verify via qPCR, western blot, and immunohistochemistry and design functional tests to further clarify the functions of these core genes in LUAD.

In summary, by integrating public databases and bioinformatics tools, this study mined four diagnostic markers (EHD2, TNS1, FHL1, and SORBS2) related to the immune infiltration in LUAD. Our study provides a new perspective on immune cell‐based development of diagnostic biomarkers that potentially contributes to the identification of diagnostic and therapeutic drugs for LUAD.

## Author Contributions

Y.Z., Q.L. and Y.C. designed and performed the research; M.N. analyzed the data; N.X. wrote the manuscript.

## Funding

The authors have nothing to report.

## Disclosure

All authors read and approved the final manuscript.

## Ethics Statement

The authors have nothing to report.

## Consent

The authors have nothing to report.

## Conflicts of Interest

The authors declare no conflicts of interest.

## Supporting Information

Additional supporting information can be found online in the Supporting Information section.

## Supporting information


**Supporting Information** Supporting Figure. 1. The expression four hub gene in the proteome data of LUAD. (A) The expression of four hub genes in the proteome of LUAD tissues and normal tissues is shown. (B) Nomogram related model analyzed four hub genes in LUAD.

## Data Availability

The data that support the findings of this study are available from the corresponding author upon reasonable request.

## References

[1] Relli V. , Trerotola M. , Guerra E. , and Alberti S. , Abandoning the Notion of Non-Small Cell Lung Cancer, Trends in Molecular Medicine. (2019) 25, no. 7, 585–594, 10.1016/j.molmed.2019.04.012. 31155338

[2] Chen J. W. and Dhahbi J. , Lung Adenocarcinoma and Lung Squamous Cell Carcinoma Cancer Classification, Biomarker Identification, and Gene Expression Analysis Using Overlapping Feature Selection Methods, Scientific Reports. (2021) 11, no. 1, 10.1038/s41598-021-92725-8. PMC8233431 34172784

[3] Zhang L. , Zhang Y. , Wang C. et al., Integrated Single-Cell RNA Sequencing Analysis Reveals Distinct Cellular and Transcriptional Modules Associated With Survival in Lung Cancer, Signal Transduction and Targeted Therapy. (2022) 7, no. 1, 10.1038/s41392-021-00824-9. PMC8758688 35027529

[4] Zhang L. , Wang S. , and Wang L. , Comprehensive Analysis Identifies YKT6 as a Potential Prognostic and Diagnostic Biomarker in Lung Adenocarcinoma, BMC Cancer. (2024) 24, no. 1, 10.1186/s12885-024-12975-3. PMC11460176 39375639

[5] Wu Q. , You L. , Nepovimova E. et al., Hypoxia-Inducible Factors: Master Regulators of Hypoxic Tumor Immune Escape, Journal of Hematology & Oncology. (2022) 15, no. 1, 10.1186/s13045-022-01292-6. PMC9166526 35659268

[6] Mou F. , Sun Q. , and Ye Z. , Advances in the Combination of Immunotherapy and Radiotherapy for Microsatellite-Stable Metastatic Colorectal Cancer, Current Proteomics. (2025) 22, no. 5, 10.1016/j.curpro.2025.100049.

[7] Christofides A. , Strauss L. , Yeo A. , Cao C. , Charest A. , and Boussiotis V. A. , The Complex Role of Tumor-Infiltrating Macrophages, Nature Immunology. (2022) 23, no. 8, 1148–1156, 10.1038/s41590-022-01267-2. 35879449 PMC10754321

[8] Zhang S. , Peng W. , Wang H. et al., C1q (+) Tumor-Associated Macrophages Contribute to Immunosuppression Through Fatty Acid Metabolic Reprogramming in Malignant Pleural Effusion, Journal for Immunotherapy of Cancer. (2023) 11, no. 8, 10.1136/jitc-2023-007441. PMC10445384 37604643

[9] Jia X. , Yan B. , Tian X. et al., CD47/SIRPalpha Pathway Mediates Cancer Immune Escape and Immunotherapy, International Journal of Biological Sciences. (2021) 17, no. 13, 3281–3287, 10.7150/ijbs.60782. 34512146 PMC8416724

[10] Park M. D. , Reyes-Torres I. , LeBerichel J. et al., TREM2 Macrophages Drive NK Cell Paucity and Dysfunction in Lung Cancer, Nature Immunology. (2023) 24, no. 5, 792–801, 10.1038/s41590-023-01475-4. 37081148 PMC11088947

[11] Yang Y. , Guo Z. , Chen W. et al., M2 Macrophage-Derived Exosomes Promote Angiogenesis and Growth of Pancreatic Ductal Adenocarcinoma by Targeting E2F2, Molecular Therapy: The Journal of the American Society of Gene Therapy. (2021) 29, no. 3, 1226–1238, 10.1016/j.ymthe.2020.11.024. 33221435 PMC7934635

[12] Tan H. , Cai M. , Wang J. et al., Harnessing Macrophages in Cancer Therapy: From Immune Modulators to Therapeutic Targets, International Journal of Biological Sciences. (2025) 21, no. 5, 2235–2257, 10.7150/ijbs.106275. 40083710 PMC11900799

[13] Li M. , Yang Y. , Xiong L. , Jiang P. , Wang J. , and Li C. , Metabolism, Metabolites, and Macrophages in Cancer, Journal of Hematology & Oncology. (2023) 16, no. 1, 10.1186/s13045-023-01478-6. PMC10367370 37491279

[14] Jiang H. , Wei H. , Wang H. et al., Zeb1-Induced Metabolic Reprogramming of Glycolysis is Essential for Macrophage Polarization in Breast Cancer, Cell Death & Disease. (2022) 13, no. 3, 10.1038/s41419-022-04632-z. PMC8897397 35246504

[15] Maes E. , Mertens I. , Valkenborg D. , Pauwels P. , Rolfo C. , and Baggerman G. , Proteomics in Cancer Research: Are We Ready for Clinical Practice?, Critical Reviews In Oncology-Hematology. (2015) 96, no. 3, 437–448, 10.1016/j.critrevonc.2015.07.006. 26277237

[16] Zhang Y. , Liu Y. , Ye Y. et al., Quantitative Proteome Analysis of Colorectal Cancer-Related Differential Proteins, Journal of Cancer Research and Clinical Oncology. (2017) 143, no. 2, 233–241, 10.1007/s00432-016-2274-5. 27659785 PMC11819047

[17] Zhu G. , Jin L. , Sun W. , Wang S. , and Liu N. , Proteomics of Post-Translational Modifications in Colorectal Cancer: Discovery of New Biomarkers, Biochimica et Biophysica Acta Reviews on Cancer. (2022) 1877, no. 4, 10.1016/j.bbcan.2022.188735. 35577141

[18] Martin-Garcia D. , Garcia-Aranda M. , and Redondo M. , Biomarker Identification Through Proteomics in Colorectal Cancer, International Journal of Molecular Sciences. (2024) 25, no. 4, 10.3390/ijms25042283. PMC10888664 38396959

[19] Lan T. , Wang J. , Qian C. , Xu X. , Zheng Q. , and Zhang Z. , Proteomic Analysis Reveals the Expression and Impact of PRDX2 in Ovarian Cancer Tissues, Current Proteomics. (2025) 22, no. 4, 10.1016/j.curpro.2025.100031.

[20] Sant P. , Rippe K. , and Mallm J. P. , Approaches for Single-Cell RNA Sequencing Across Tissues and Cell Types, Transcription. (2023) 14, no. 3-5, 127–145, 10.1080/21541264.2023.2200721. 37062951 PMC10807473

[21] Sun Y. , Liu Z. , Fu Y. et al., Single-Cell Multi-Omics Sequencing and Its Application in Tumor Heterogeneity, Briefings in Functional Genomics. (2023) 22, no. 4, 313–328, 10.1093/bfgp/elad009. 37078714

[22] Wang H. , Zhao T. , Zeng J. et al., Methods and Clinical Biomarker Discovery for Targeted Proteomics Using Olink Technology, Proteomics Clinical Applications. (2024) 18, no. 5, 10.1002/prca.202300233. 38726756

[23] Zhu C. , Chen Z. , Wang S. , Cao J. , Cheng Y. , and Zheng M. , Single-Cell Analyzing of Tumor Microenvironment and Cell Adhesion Between Early and Late-Stage Lung Cancer, Molecular Immunology. (2024) 171, 1–11, 10.1016/j.molimm.2024.04.013. 38696904

[24] Zhang N. , Tian H. , Huang Q. , Liu A. , and Zhang F. , Investigating the Mechanism of Curcumin Against LUAD Malignant Progression and Macrophage M2 Polarization Based on Network Pharmacology, Bioinformatics, and Molecular Docking, Letters in Drug Design & Discovery. (2025) 22, no. 8, 10.1016/j.lddd.2025.100140.

[25] Manyuan L. , Xufeng D. , Dong Z. , Xiaoqing L. , Jigang D. , and Quanxing L. , A Novel Methylation-Based Model for Prognostic Prediction in Lung Adenocarcinoma, Current Genomics. (2024) 25, no. 1, 26–40. 38544827 10.2174/0113892029277397231228062412 PMC10964088

[26] Lindberg I. , Saleh A. , Tutzauer J. et al., Prognostic Relevance of CD163(+) Immune Cells in Patients With Metastatic Breast Cancer, Cancer Immunology, Immunotherapy: CII. (2025) 74, no. 2, 10.1007/s00262-024-03892-2. PMC11699000 39751847

[27] Creighton C. J. , Clinical Proteomics Towards Multiomics in Cancer, Mass Spectrometry Reviews. (2024) 43, no. 6, 1255–1269, 10.1002/mas.21827. 36495097

[28] Gheybi E. , Hosseinzadeh P. , Tayebi-Khorrami V. , Rostami M. , and Soukhtanloo M. , Proteomics in Decoding Cancer: A Review, Clinica Chimica Acta; International Journal of Clinical Chemistry. (2025) 574, 10.1016/j.cca.2025.120302. 40220985

[29] Luo S. , Peng H. , Shi Y. et al., Integration of Proteomics Profiling Data to Facilitate Discovery of Cancer Neoantigens: A Survey, Briefings in Bioinformatics. (2025) 26, no. 2, 10.1093/bib/bbaf087. PMC11886573 40052441

[30] Song J. and Yang H. , Identifying New Biomarkers and Potential Therapeutic Targets for Breast Cancer Through the Integration of Human Plasma Proteomics: A Mendelian Randomization Study and Colocalization Analysis, Frontiers in Endocrinology. (2024) 15, 10.3389/fendo.2024.1449668. PMC11439655 39351539

[31] Wang K. , Yi H. , Wang Y. , Jin D. , Zhang G. , and Mao Y. , Proteome-Wide Multicenter Mendelian Randomization Analysis to Identify Novel Therapeutic Targets for Lung Cancer, Archivos de Bronconeumología. (2024) 60, no. 9, 553–558, 10.1016/j.arbres.2024.05.007. 38824092

[32] Taranto D. , Kloosterman D. J. , and Akkari L. , Macrophages and T Cells in Metabolic Disorder-Associated Cancers, Nature Reviews Cancer. (2024) 24, no. 11, 744–767, 10.1038/s41568-024-00743-1. 39354070

[33] Chaudhary A. , Patil P. , Raina P. , and Kaul-Ghanekar R. , Matairesinol Repolarizes M2 Macrophages to M1 Phenotype to Induce Apoptosis in Triple-Negative Breast Cancer Cells, Immunopharmacology and Immunotoxicology. (2025) 47, no. 1, 8–22, 10.1080/08923973.2024.2425028. 39722605

