# Supplementary material for: Screening Potential Diagnostic Biomarkers of Lung Adenocarcinoma Related to Immune Infiltration Based on Single‐Cell Proteomic Integration Analysis
Source: Can Respir J. 2026 Aug 17;2026:7004493. doi: 10.1155/carj/7004493 (PMC13482034; doi:10.1155/carj/7004493)
Supplement: Supplementary file 1 [file CARJ-2026-7004493-s001.pdf]

(A)

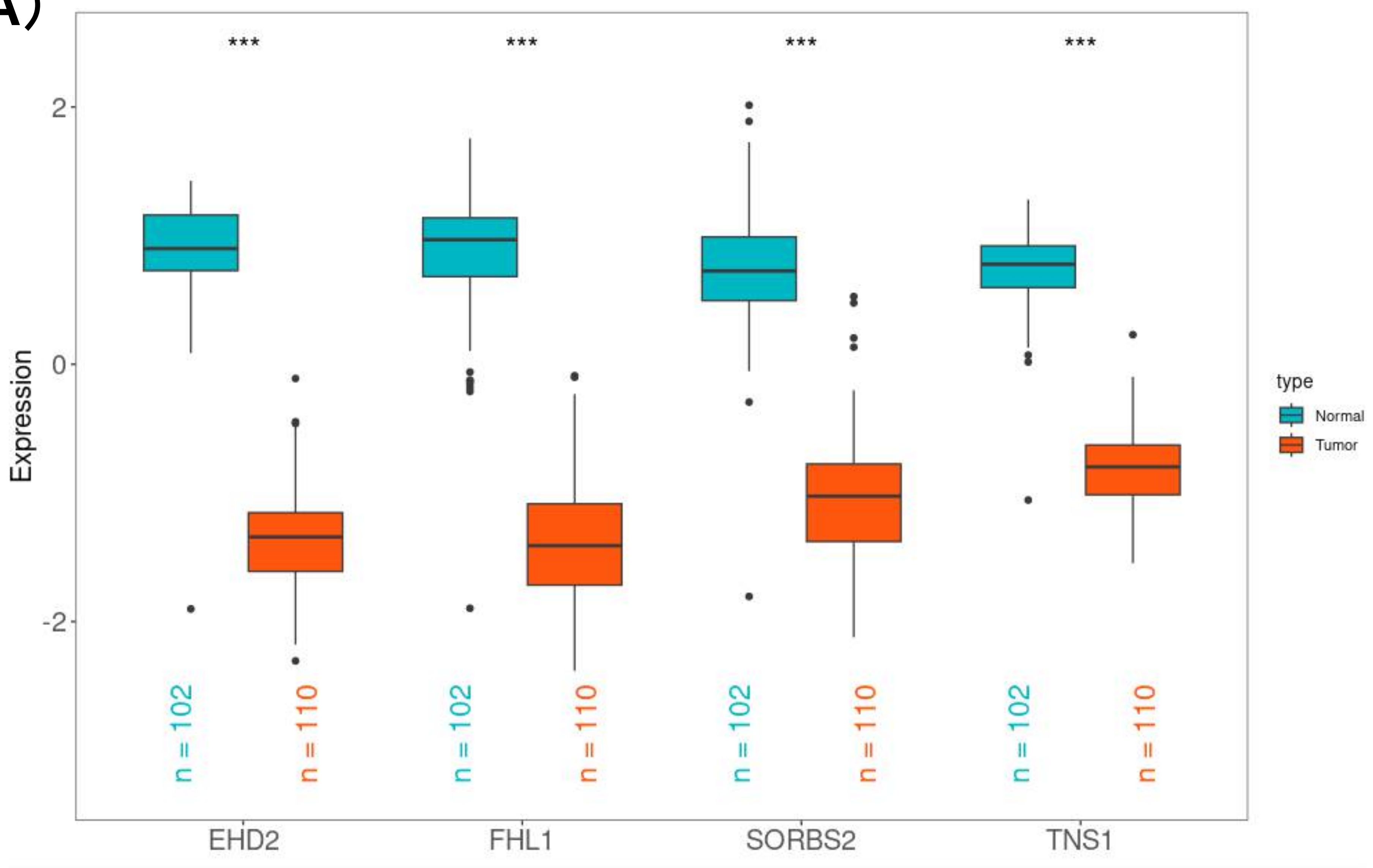

***
***
***
***
2
0
-2
Expression
type
Normal
Tumor
n = 102
n = 110
n = 102
n = 110
n = 102
n = 110
n = 102
n = 110
EHD2
FHL1
SORBS2
TNS1

(B)

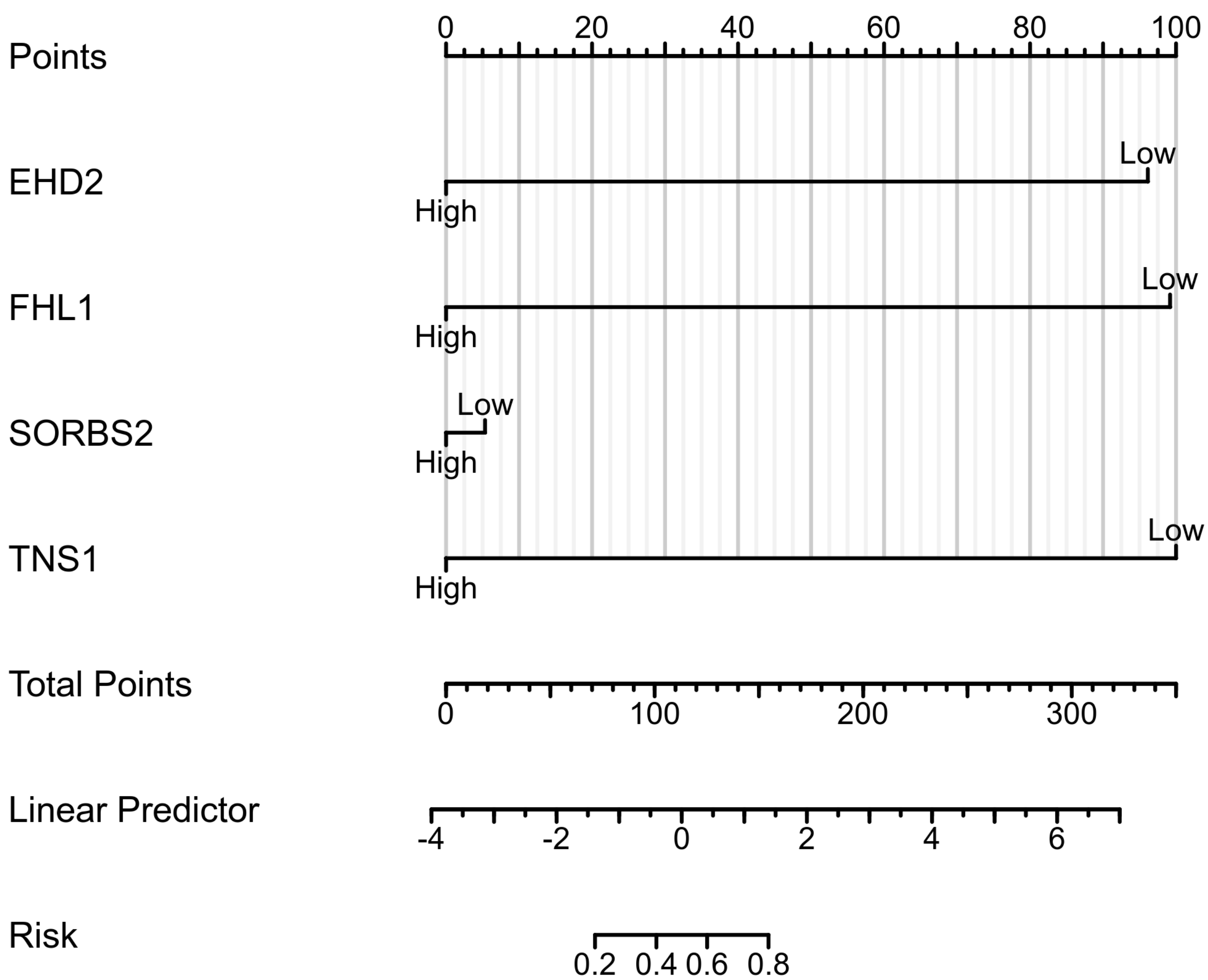

Points
0
20
40
60
80
100
EHD2
Low
High
FHL1
Low
High
SORBS2
Low
High
TNS1
Low
High
Total Points
0
100
200
300
Linear Predictor
-4
-2
0
2
4
6
Risk
0.2
0.4
0.6
0.8
